# Digital versus non-digital health interventions to improve iron supplementation in pregnant women: a systematic review and meta-analysis

**DOI:** 10.3389/fmed.2024.1375622

**Published:** 2024-05-30

**Authors:** Yu Shao, Chao Meng, Ying-Zhi Liang

**Affiliations:** Department of Maternal Health, Beijing Haidian Maternal and Child Health Hospital, Beijing, China

**Keywords:** digital health, medication adherence, iron deficiency, systematic review, pregnancy

## Abstract

**Objective:**

To investigate the effects of digital health interventions for improving adherence to oral iron supplementation in pregnant women.

**Literature search:**

Five databases were searched from their inception to October 2023 with no date restrictions.

**Study selection:**

Randomized controlled trials (RCTs) that assessed the effects of digital health interventions on adherence to oral iron supplementation (e.g., tablets and capsules) compared to non-digital health interventions for pregnant women were eligible.

**Data synthesis:**

We calculated standardized mean differences (SMDs) and mean differences (MDs) with 95% confidence intervals (CIs) for continuous variables using the inverse variance method. We calculated odds ratios (OR) with 95%CI for categorical variables using the Mantel–Haenszel model. The certainty of the evidence was assessed using the Grading of Recommendations Assessment, Development, and Evaluation (GRADE) approach. The risk of bias of the included RCTs was assessed using the Cochrane risk of bias tool 2.0.

**Results:**

Ten trials with 1,633 participants were included. Based on 7 trials, digital health interventions can improve objective adherence rate comparing with non-digital health interventions (1,289 participants, OR = 4.07 [2.19, 7.57], *p* < 0.001, I^2^ = 69%) in pregnant women. Digital health interventions can improve subjective adherence behavior comparing with non-digital health interventions (3 trials, 434 participants, SMD = 0.82 [0.62, 1.01], *p* < 0.001, I^2^ = 0%) in pregnant women. Based on 3 trials, digital health interventions can improve tablets consumption comparing with non-digital health interventions (333 participants, SMD = 1.00 [0.57, 1.42], *p* < 0.001, I^2^ = 66%) in pregnant women. Digital health interventions can improve hemoglobin level comparing with non-digital health interventions (7 trials, 1,216 participants, MD = 0.59 [0.31, 0.88], *p* < 0.001, I^2^ = 93%) in pregnant women.

**Conclusion:**

Digital health interventions were effective at improving adherence to oral iron supplementation and hemoglobin levels in pregnant women.

## 1 Introduction

Iron deficiency is the most common pathologic cause of anaemia in pregnancy, which is a prevalent global health problem associated with adverse maternal and fetal outcomes (1, 2). The World Health Organization (WHO) estimates that over 40 percent of pregnancies are complicated by anaemia (3, 4). The recommended intervention for iron deficiency is oral iron supplementation (5–7), which is safe, cost-effective, easily accessible, and effective if tolerated. Considerable evidence suggests that iron supplementation reduces anaemia and iron deficiency during pregnancy (4, 8–10). However, ensuring optimal adherence to prescribed iron regimens remains a critical factor affecting its effectiveness (11, 12).

The WHO defined adherence as “the extent to which a patient’s behavior matches the agreed recommendations from a healthcare provider (13, 14).” Several strategies improve adherence to oral iron supplementation in pregnant women, such as health-care professional training, individual counselling or group education sessions, financial incentives, and family/peer support (15, 16). Nevertheless, the sustained impact of these intervention strategies is limited, with only a modest effect (11, 17–19). Innovative, engaging, and sustainable techniques are imperative to enhance adherence to oral iron supplementation. In response to this challenge, digital health interventions have emerged as a promising tool to improve adherence in various healthcare settings.

Digital health interventions (20, 21), including mobile applications, telehealth platforms and wearable devices, represents an innovative approach to addressing this issue. Digital health interventions have been shown to help pregnant women better manage health issues during pregnancy, increase awareness of changes during pregnancy and intervene in time to address potential risks (22, 23). These interventions have the potential to provide personalised support, timely reminders, and educational resources to pregnant women to improve their adherence to oral iron supplementation. Understanding the role of digital health in promoting adherence to iron supplementation during pregnancy is critical to advancing healthcare practices and ensuring the well-being of both mothers and infants (24, 25). Nevertheless, there is no review synthesising the latest clinical evidence on the effectiveness of digital health interventions in promoting adherence to oral iron supplementation in pregnant women.

Therefore, this systematic review and meta-analysis aims to comprehensively assess the effectiveness of digital health interventions on adherence to oral iron supplementation in pregnant women.

## 2 Methods

The review is reported according to the Preferred Reporting Items for Systematic Reviews and Meta-Analyses (PRISMA) statement (26), and follows the Cochrane Handbook for Systematic Reviews of Interventions, Version 6.3 (27). The protocol was registered in the International Prospective Register of Systematic Reviews (PROSPERO): CRD42024498830.

### 2.1 Electronic searches

We searched PubMed, Embase, The Cochrane Library, Web of Science, and Scopus until October 2023 with no date restrictions. The search strategy was developed with the following key terms: pregnant women, digital health intervention, adherence, and oral iron supplementation (see Supplementary Table S1) for the full search strategy). The manual search of references was conducted in addition to the electronic database search to identify potentially eligible records.

### 2.2 Selection criteria

Studies were selected independently by two authors using predefined criteria. Disagreements were resolved by consensus. In cases where the full manuscript was not available, the author was contacted by e-mail. Randomized controlled trials (RCTs) investigating oral iron supplementation (e.g., tablets and capsules) adherence in digital health interventions compared with non-digital health interventions for pregnant women were eligible. Pregnant women of any gestational age and parity were eligible. Digital health interventions are interventions delivered through digital technologies such as text messages, phone calls, websites, and apps. Previous studies (28, 29) have shown that results between subjective and objective measures of adherence are not always consistent, and the study by Cross et al. (14) categorized adherence measures into subjective and objective measures. Therefore, the primary outcomes assessed adherence to oral iron supplementation (with objective and/or subjective measure) and tablets consumption. The objective measure is objective adherence rate, i.e., the proportion of people with high adherence (calculated from used/unused prescription pill count). The subjective measure is subjective adherence behavior, i.e., the participants’ self-reported behavior questionnaire. The tablets consumption is quantified by calculating total number of tablets taken per week. The secondary outcomes assessed the effects of oral iron supplementation with digital health interventions using hemoglobin level (g/dL). Trials conducted on animals were excluded. Trials not available in English were excluded. No date restrictions were used.

### 2.3 Data extraction

Data from the included studies were independently extracted by 2 authors guided by Cochrane handbook (27). Any disagreement was resolved by discussion as required. The following descriptive data were extracted: author, country of study, published year, participant characteristics (sample size, age, and gender), intervention characteristics, and outcome measures. The primary outcome measures were objective adherence rate, subjective adherence behavior, and tablets consumption. The secondary outcome measure was hemoglobin level. We extracted immediate post-intervention data as primary outcome data for meta-analyses.

### 2.4 Data analysis

We used random-effects models for all meta-analyses due to the expected heterogeneity between the studies. We calculated standardized mean differences (SMDs) and mean differences (MDs) with 95% confidence intervals (CIs) for continuous variables with the inverse variance method. We calculated odds ratio (OR) and 95% confidence intervals (CI) for category variables with the Mantel–Haenszel model. SDs were calculated from available data (eg, 95% CI or *p* value) following the Cochrane guidelines where necessary (27, 30). The effect size was interpreted as small (0.2), moderate (0.5), or large (0.8). Statistical heterogeneity was assessed using the I^2^, with classification as low (I^2^ < 25%), moderate (I^2^ = 25–50%), substantial (I^2^ = 50–75%), and considerable (I^2^ > 75%) (27). Publication bias was assessed through visual inspection of funnel plot, if a sufficient number of studies was included in the analysis (31). We planned no subgroup analyses due to the limited number of studies. We conducted sensitivity analyses by omitting studies one by one in order to assess the robustness of meta-analyses’ results. We used Review Manager (version 5.4.1) to perform all statistical analyses.

Two reviewers independently conducted an evaluation of the methodological quality of the original articles using the Cochrane risk of bias tool (version 2, ROB2) (32). This tool assesses seven potential sources of bias across five domains, including randomization process, intended interventions, missing outcome data, measurement of the outcome, and selection of the reported result. Each trial underwent assessment in these five bias domains, resulting in a summary risk-of-bias score for each domain and an overall classification (low risk, some concerns, or high risk of bias). Any disagreements were addressed through discussion or adjudication. The outcomes from these assessments were visualized and analyzed using an Excel RoB2 tool (32).

The quality of evidence for each outcome was evaluated based on the Grading of Recommendations Assessment, Development, and Evaluation (GRADE) guidelines (33). Given that only Randomized Controlled Trials (RCTs) were included, each outcome was initially assigned a high level of certainty. Two reviewers conducted the assessment of evidence quality using the GRADE system, and any potential disagreements were addressed through discussion.

## 3 Results

### 3.1 Study selection and participants’ characteristics

**Figure 1:**
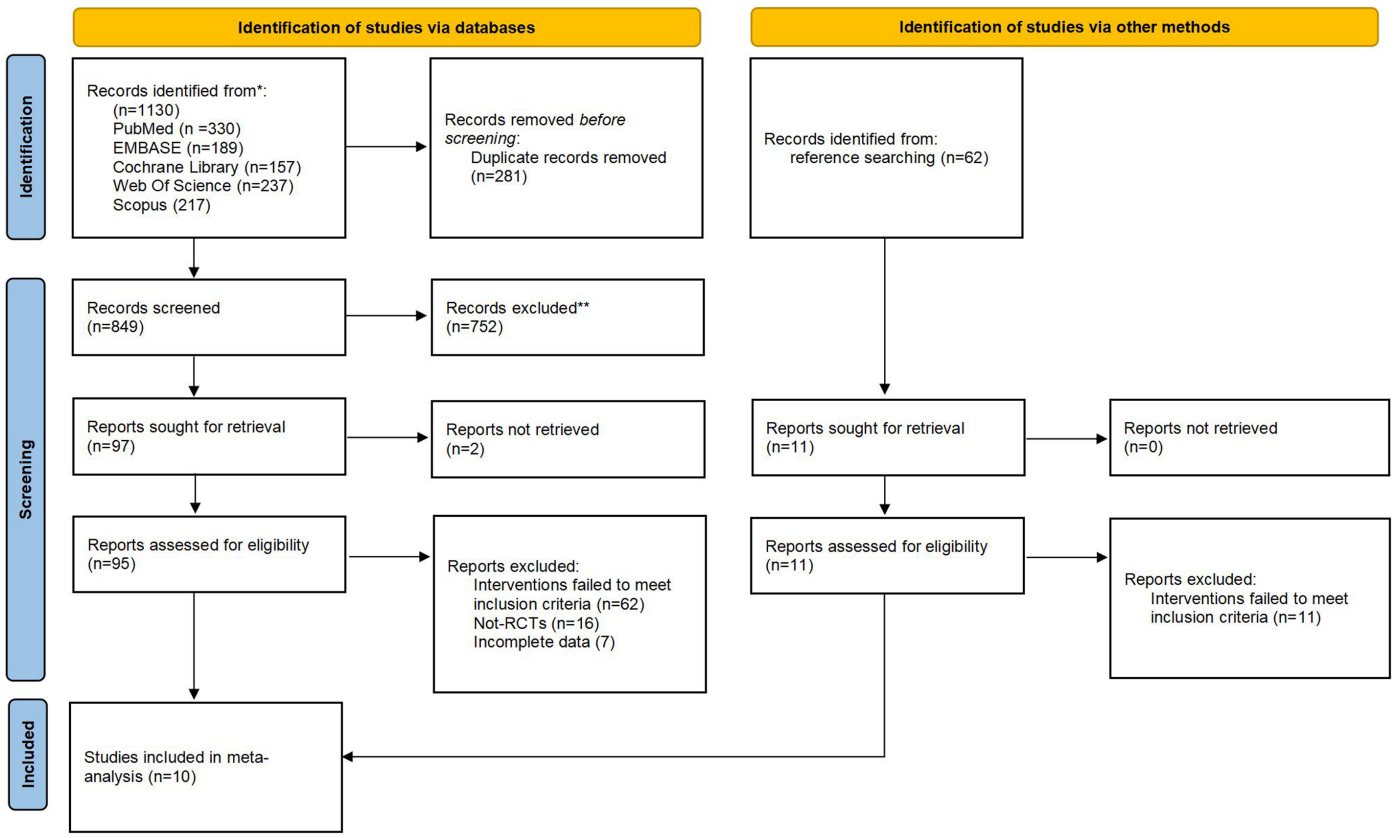
Flowchart of the study selection process.

The preliminary database search yielded 1,130 citations (Figure 1). After removing duplicate records, 849 records remained. After reviewing the titles and abstracts, 752 records were excluded as they failed to meet the eligibility criteria. Ninety-five full texts were then screened, resulting in the exclusion of 85 studies. Of the excluded studies, 62 were excluded due to an inappropriate intervention, 16 due to an incorrect study design and 7 due to incomplete data. A manual search did not identify any further articles. Finally, 10 trials with 1,633 participants were considered eligible for inclusion.

Descriptive characteristics of the 10 included trials are detailed in Table 1. Trials were published from 2014 to 2023 containing 1,633 participants (mean age: 24.3 to 29.6 years). Three trials were conducted in India (34–36), with the remainder in Indonesia (37), Saudi Arabia (38), Ethiopia (39), Indonesia (40), Malaysia (41), Jordan (42), Ahvaz (43). The intervention during for the included trials were 4 to 12 weeks, participants were followed up immediately postintervention. There is significant variation in the elements of the digital health interventions. Five trials conducted digital health interventions with WhatsApp (36–38, 40, 42), four with SMS (35, 36, 39, 43), two with phone call (34, 36), and one with web-based education program (41). Almost all the trials were multi-component interventions. All trials except two (40, 42) delivered the intervention components of reminder. Furthermore, all trials but one (34) had an education intervention component. Three trials assessed adherence through different subjective adherence behavior measures using Medical Adherence Rating Scale (MARS-5) (35), Perceived behavior control score (41), and Compliance check list (42).

**Table 1:** Characteristics of included studies.

Author (year) country	Sample characteristics *N*; Age;		Description of intervention	Description of control	Outcomes measures, follow-up (weeks)
	Intervention group	Control group			
Sharma (2023) India	127; 24.3 (3.9)	123; 24.8 (4.1)	Telephonic intervention for 4 weeks comprising of text reminders, whatsapp audio messages and telephonic calls.	Usual care.	Objective adherence rate Hemoglobin level.
Ramachandran (2023) India	59; NA	58; NA	Video-assisted nutrition education, leaflet distribution, and SMS alert twice a day for four weeks.	Usual care.	Objective adherence rate Medical Adherence Rating Scale (MARS-5) Hemoglobin level.
Arifah (2023) Indonesia	22; 28.04	22; 27.31	Daily educational and reminder messages through whatsapp through personal chat.	Standard antenatal care and health information.	Tablets consumption.
Sontakke (2022) India	120; 26.8 (4.48)	120; 26.54 (4.42)	Standard therapy following the study guidelines with the addition of fortnightly mobile phone call reminders.	Standard therapy.	Hemoglobin level Objective adherence rate.
Elsharkawy (2022) Saudi Arabia	98; 25.76 (5.42)	98; 26.10 (5.19)	One educational health message and four reminders in Arabic every week through the whatsapp platform.	Usual care.	Hemoglobin level Tablets consumption Objective adherence rate.
Berhane (2022) Ethiopia	122; 28.1 (5.19)	122; 28.1 (5.19)	Brochure with take-home key message (in local languages); Counselling and discussion on uptake of the IFAS and risk factors of NTDs and other adverse pregnant and birth outcome as well as methods of preventing them; Reminding SMS text messages via mobile phone.	Usual care without preconception picture-based education and counselling.	Objective adherence rate.
Abd Rahman (2022) Malaysia	54; 28.27 (4.411)	50; 28.72 (5.639)	During week 1, each video was disseminated daily for 6 days, followed by a weekly reminder in weeks 2 to 5. These changes were made because of logistic problems and practicality. We used whatsapp to deliver the videos to the participants in the intervention group. Each video session lasted for approximately 3to 5 min.	Usual care.	Hemoglobin level Perceived behavior control score.
Ariyani (2022) Indonesia	71; 27 (5.47)	74; 27 (5.47)	A web application-based antenatal education program.	A face-to-face antenatal education program.	Objective adherence rate.
Abujilban (2019) Jordan	100; 28.4 (6.61)	100; 29.6 (6.61)	Received individualized teaching and the video from the researcher via the whatsapp application on their smart phones.	Standard care.	Hemoglobin level Compliance check list.
Khorshid (2014) Ahvaz	49; 24.7 (4.2)	44; 25.5 (6.6)	From the 16th week of pregnancy, three reminders and four educational health messages were sent to participants in the intervention group every week for 12 weeks.	Usual care.	Hemoglobin level Tablets consumption objective adherence rate.

NA, not available.

### 3.2 Risk-of-bias assessment

**Figure 2:**
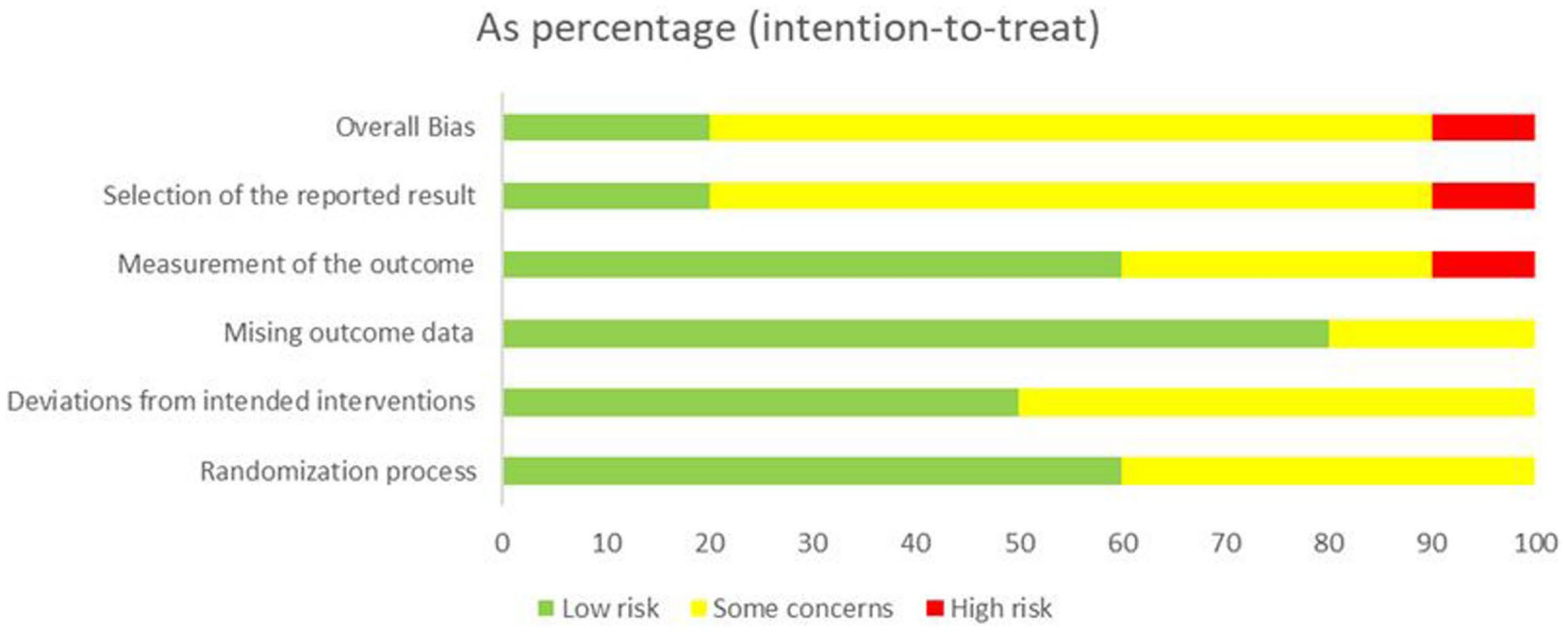
Risk of bias for all included studies.

**Figure 3:**
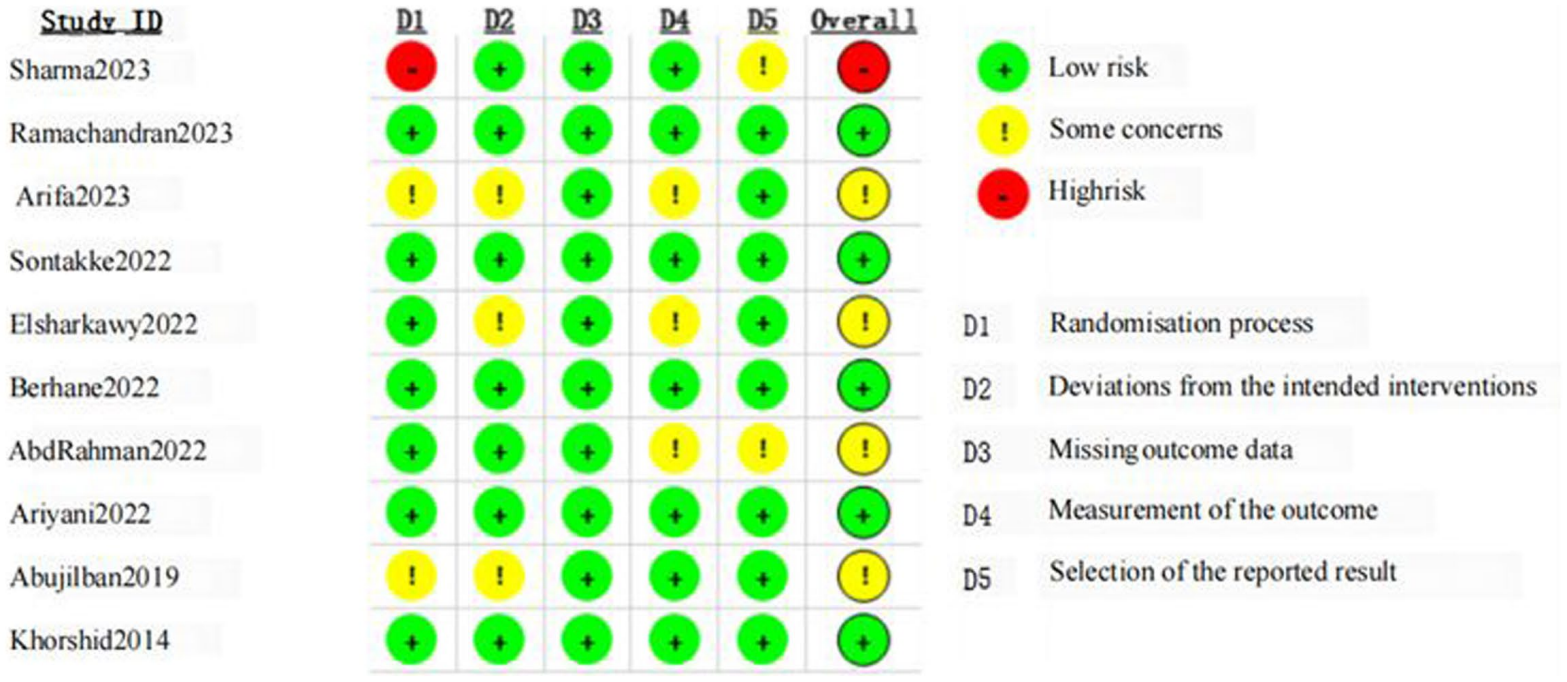
Summary of distribution of different biases.

Details of the risk of bias assessment are provided in Figures 2, 3. The assessment resulted in high risk of bias in 1 trial (36), some concerns of bias in 4 trials (37, 38, 41, 42), and low risk of bias in 5 trials (34, 35, 39, 40, 43). Due to insufficient information, almost half of trials were scored unclear risk in the randomization process or/and deviations from the intended interventions. Most of the trials were unclear or high risk of the selection of the reported result because of the lacked a pre-registered protocol, with only three studies reporting a registered protocol for the clinical trial (36, 38, 39).

### 3.3 Effects of interventions

The GRADE summary of findings for the outcomes is reported in Table 2.

**Table 2:** The GRADE summary of findings for the outcomes.

Digital health interventions compared to non-digital interventions for pregnant women					
Outcomes	Anticipated absolute effects^*^ (95% CI)		Relative effect (95% CI)	№ of participants (studies)	Certainty of the evidence (GRADE)
	Risk with non-digital	Risk with digital			
Objective adherence rate	557 per 1,000	836 per 1,000 (732 to 906)	OR 4.07 (2.17 to 7.65)	1,289 (7 RCTs)	⨁⨁⨁◯ Moderate^a^
Subjective adherence behavior	–	SMD 0.82 higher (0.62 higher to 1.01 higher)	–	434 (3 RCTs)	⨁⨁⨁⨁ High
Tablets consumption	–	SMD1 higher (0.57 higher to 1.42 higher)	–	333 (3 RCTs)	⨁⨁⨁◯ Moderate^a^
Hemoglobin level	–	MD 0.59 higher (0.31 higher to 0.88 higher)	–	1,216 (7 RCTs)	⨁⨁◯◯ Low^b^

^*^The risk in the intervention group (and its 95% confidence interval) is based on the assumed risk in the comparison group and the relative effect of the intervention (and its 95% CI). CI, confidence interval; MD, mean difference; OR, odds ratio; SMD, standardized mean difference. GRADE Working Group grades of evidence. High certainty: we are very confident that the true effect lies close to that of the estimate of the effect. Moderate certainty: we are moderately confident in the effect estimate: the true effect is likely to be close to the estimate of the effect, but there is a possibility that it is substantially different. Low certainty: our confidence in the effect estimate is limited: the true effect may be substantially different from the estimate of the effect. Very low certainty: we have very little confidence in the effect estimate: the true effect is likely to be substantially different from the estimate of effect.

a Evidence of considerable inconsistency (heterogeneity in the I^2^test > 50%).

b Evidence of serious inconsistency (heterogeneity in the I^2^test > 75%).

### 3.4 Objective adherence rate

**Figure 4:**
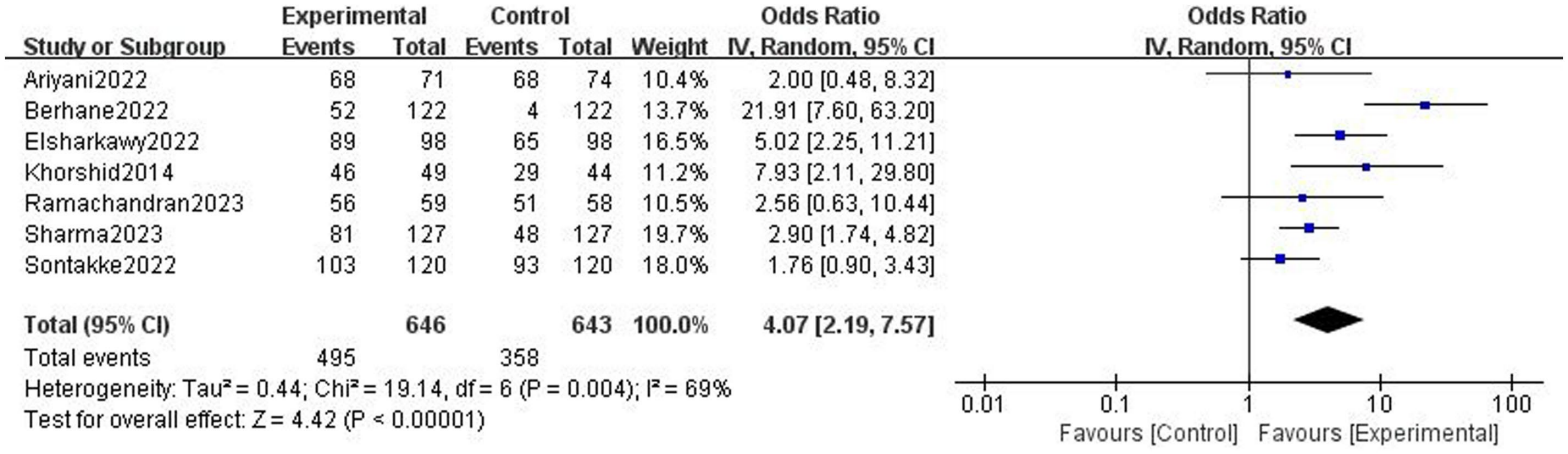
Effect of digital health interventions on the objective adherence rate.

Based on 7 trials, there was moderate-certainty evidence that digital health interventions can improve objective adherence rate comparing with non-digital health interventions (1,289 participants, OR = 4.07 [2.19, 7.57], *p* < 0.001, I^2^ = 69%, Figure 4) in pregnant women. If we excluded the study of Berhane et al. (39) the I^2^ decreased to 23% and the results remained unchanged (1,045 participants, OR = 2.98 [1.99, 4.46], *p* < 0.001, I^2^ = 23%). The funnel plot of objective adherence rate was almost visually symmetrical, indicating that there is no obvious publication bias (Supplementary Figure S1).

### 3.5 Subjective adherence behavior

**Figure 5:**
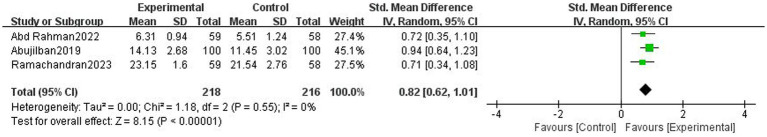
Effect of digital health interventions on the subjective adherence behavior.

There was high-certainty evidence that digital health interventions can improve subjective adherence behavior comparing with non-digital health interventions (3 trials, 434 participants, SMD = 0.82 [0.62, 1.01], *p* < 0.001, I^2^ = 0%, Figure 5) in pregnant women.

### 3.6 Tablets consumption

**Figure 6:**
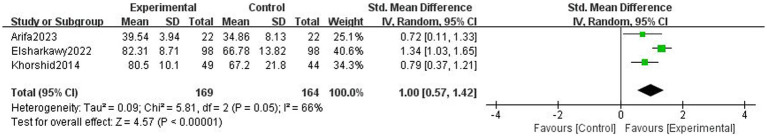
Effect of digital health interventions on the tablets consumption.

Based on 3 trials, there was moderate-certainty evidence that digital health interventions can improve tablets consumption comparing with non-digital health interventions (333 participants, SMD = 1.00 [0.57, 1.42], *p* < 0.001, I^2^ = 66%, Figure 6) in pregnant women.

### 3.7 Hemoglobin level

**Figure 7:**
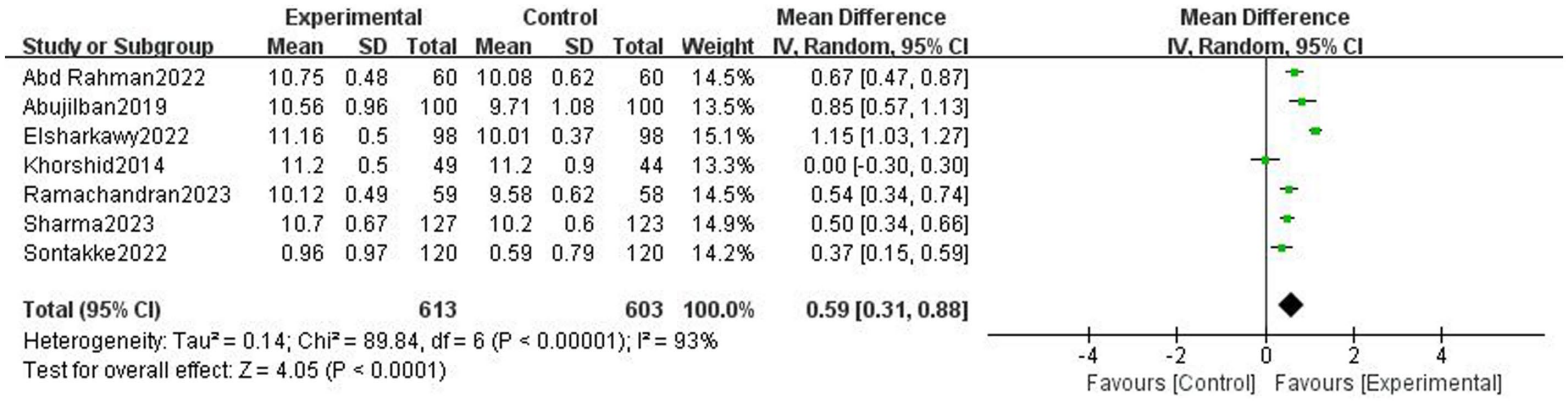
Effect of digital health interventions on the hemoglobin level.

There was low-certainty evidence that digital health interventions can improve hemoglobin level comparing with non-digital health interventions (7 trials, 1,216 participants, MD = 0.59 [0.31, 0.88], *p* < 0.001, I^2^ = 93%, Figure 7) in pregnant women. If we excluded the study of Elsharkawy et al. (38) the I^2^ decreased to 76% and the results remained unchanged (1,020 participants, MD = 0.50 [0.31, 0.68], *p* < 0.001, I^2^ = 76%). The funnel plot of hemoglobin level was basically symmetrical, suggesting no significant publication bias (Supplementary Figure S2).

## 4 Discussion

This systematic review and meta-analysis found that, based on low- to high-quality evidence, digital health interventions were effective in improving adherence to oral iron supplementation and hemoglobin levels in pregnant women. There was high level of evidence that digital health interventions can improve the subjective adherence behavior of pregnant women compared to conventional treatment. On the basis of a moderate level of evidence, digital health interventions can improve the objective adherence rate and tablet consumption of pregnant women compared to conventional treatment. Digital health interventions can improve the hemoglobin level of pregnant women compared to conventional treatment with low level of evidence. Sensitivity analyses did not alter any results, indicating robust main findings. In the future, digital health interventions can be considered as a routine approach to improve adherence to oral iron supplementation in pregnant women. By adopting this strategy, we expect to substantially improve the effectiveness of oral iron supplementation in preventing anaemia in pregnant women.

There was considerable heterogeneity in the results of the meta-analyses of the objective adherence rate and hemoglobin level. The heterogeneity in the objective adherence rate may be partly explained by the study by Berhane et al. (39). When this study was excluded, the heterogeneity was reduced to I^2^ = 23%. Differing from other studies with simple digital technology intervention, the intervention of Berhane et al. was a multidimensional digital health intervention including picture-based health education and counselling and SMS reminders. The high heterogeneity in hemoglobin levels resulted from several factors, though excluding the study by Elsharkawy et al. (38). Significantly reduced the heterogeneity to I^2^ = 76%, but it was still highly heterogeneous. The baseline hemoglobin levels of participants in each trial were variable, and the doses of oral iron supplementation prescribed were also different, all of which contributed to the high clinical heterogeneity of post-intervention hemoglobin levels between trials. Furthermore, due to the limited number of studies, visual inspection of funnel plots should be interpreted with caution when determining publication bias.

To our knowledge, this is the first systematic review to evaluate the efficacy of digital health interventions on adherence to oral iron supplementation in pregnant women. A previous review (11) by Gomes et al. investigated the efficacy of interventions to increase adherence on micronutrient supplementation during pregnancy. The review found that most education-based interventions, consumption monitoring by volunteer health workers or family members, text message reminders, free provision of supplements, community-mobilized multi-component interventions and participatory action research interventions improved adherence. However, no meta-analyses were carried out due to the limited number of trials available. Since the publication of this review in 2021, numerous RCTs (34–41) examining digital health interventions on adherence to oral iron supplementation have been published. Furthermore, this review found that different digital intervention components may have variable effects on oral iron supplementation adherence. Ariyani et al. (40) showed that when there was no reminder component in the intervention, it may reduce the objective adherence rate (OR: 2.00 [0.48, 8.32]) compared with the pooled OR (4.07 [2.17, 7.65]). Additionally, Sontakke et al. (34) demonstrated that objective adherence rates may also be reduced when there was no educational component in the intervention (OR: 1.76 [0.90, 3.43]). However, due to the insufficient number of studies, no subgroup analyses could be conducted by intervention type (reminder/non-reminder or education/non-education). More relevant studies are needed in the future to explore which digital intervention programs are the most effective in enhancing adherence to oral iron supplementation.

Several limitations of this systematic review and meta-analysis are worth highlighting. Firstly, due to the limited number of studies included in each meta-analysis, publication bias tests and subgroup analyses could not be conducted. Second, the results of the meta-analyses of objective adherence and hemoglobin levels were highly heterogeneous. Also, the number of studies on subjective adherence was limited, and future studies on the subjective adherence behavior of pregnant women should be strengthened. In addition, there are different subjective adherence behavior scales across studies (35, 41, 42), and no uniformly standardized subjective adherence behavior scales are currently available. Finally, the trials included in this review all evaluated the effectiveness of adherence immediately post-intervention and lacked long-term adherence outcomes.

It is noteworthy that future research could further explore the benefits of different digital technologies in improving adherence to oral iron supplementation in pregnant women (44). This investigation could include, but is not limited to, mobile applications, smart devices, remote monitoring systems, among others. By comparing the effectiveness of different digital technologies, we can gain a more comprehensive understanding of which tools are most effective for different subgroups of pregnant women, allowing for personalised optimisation of digital health interventions to meet the specific needs of diverse cohorts of people (45, 46). These tailored intervention approaches promise to play a crucial role in future maternal health management, providing more effective clinical support to improve adherence to oral iron supplementation and prevent anaemia. However, the clinical application of digital health interventions in maternal health still faces challenges such as privacy, data security and cultural differences (47–49). In addition, more large-scale studies are needed to validate the effectiveness and sustainability of these technologies.

## 5 Conclusion

There was low- to high-quality evidence supporting digital health interventions for improving adherence to oral iron supplementation and hemoglobin levels in pregnant women. Clinicians could consider digital health interventions to improve adherence to oral iron supplementation in pregnant women, potentially enhancing the effects of oral iron supplementation.

## Data availability statement

The original contributions presented in the study are included in the article/Supplementary material, further inquiries can be directed to the corresponding author.

## Author contributions

YS: Conceptualization, Investigation, Writing – original draft, Writing – review & editing, Supervision. CM: Conceptualization, Data curation, Formal analysis, Investigation, Methodology, Software, Writing – original draft, Writing – review & editing. YZ-L: Conceptualization, Investigation, Writing – original draft, Writing – review & editing, Data curation, Formal analysis, Methodology, Software.

## Supplementary material

SUPPLEMENTARY FIGURE S1 Funnel plot of publication bias for objective adherence rate.

SUPPLEMENTARY FIGURE S2 Funnel plot of publication bias for hemoglobin level.

## References

[1] Ruiz de Viñaspre-Hernández R Gea-Caballero V Juárez-Vela R Iruzubieta-Barragán FJ. The definition, screening, and treatment of postpartum anemia: a systematic review of guidelines. Birth. (2021) 48:14–25. doi: 10.1111/birt.12519, PMID: 33274766

[2] Reveiz L Gyte GM Cuervo LG Casasbuenas A. Treatments for iron-deficiency anaemia in pregnancy. Cochrane Database Syst Rev. (2011) 10:CD003094. doi: 10.1002/14651858.CD003094.pub3 PMC12989263 21975735

[3] Indicator Metadata Registry Details . Available at: https://www.who.int/data/gho/indicator-metadata-registry/imr-details/4552 (Accessed January 22, 2024).

[4] GHO | By category | Prevalence of anaemia in pregnant women – Estimates by WHO region. WHO. Available at: https://apps.who.int/gho/data/view.main.ANAEMIAWOMENPWREG (Accessed January 22, 2024).

[5] Singh RB Mishra S Kumar S Tiwari AM De Meester F Goyal RK . Micronutrient formulations for prevention of complications of pregnancy. Front Biosci (Schol Ed). (2018) 10:175–84. doi: 10.2741/s507, PMID: 28930525

[6] Tiwari AKM Mahdi AA Mishra S. Assessment of liver function in pregnant anemic women upon oral iron and folic acid supplementation. J Gynecol Obstet Hum Reprod. (2018) 47:45–9. doi: 10.1016/j.jogoh.2017.11.010, PMID: 29196155

[7] Tiwari AKM Mahdi AA Mishra S Parveen H Fatima G. Effect of iron and folate supplementation on Pb levels in pregnant anemic women: a prospective study. Free Radic Res. (2020) 54:662–9. doi: 10.1080/10715762.2020.1825704, PMID: 32954897

[8] Bradley R Lakpa KL Burd M Mehta S Katusic MZ Greenmyer JR. Fetal alcohol Spectrum disorder and Iron homeostasis. Nutrients. (2022) 14:4223. doi: 10.3390/nu14204223, PMID: 36296909 PMC9607572

[9] Helfrich KK Saini N Kling PJ Smith SM. Maternal iron nutriture as a critical modulator of fetal alcohol spectrum disorder risk in alcohol-exposed pregnancies. Biochem Cell Biol. (2018) 96:204–12. doi: 10.1139/bcb-2017-0206, PMID: 29017023 PMC5914169

[10] Tiwari AKM Mahdi AA Chandyan S Zahra F Godbole MM Jaiswar SP . Oral iron supplementation leads to oxidative imbalance in anemic women: a prospective study. Clin Nutr. (2011) 30:188–93. doi: 10.1016/j.clnu.2010.08.001, PMID: 20888091

[11] Gomes F King SE Dallmann D Golan J da Silva ACF Hurley KM . Interventions to increase adherence to micronutrient supplementation during pregnancy: a systematic review. Ann N Y Acad Sci. (2021) 1493:41–58. doi: 10.1111/nyas.14545, PMID: 33400303 PMC8169578

[12] Saragih ID Dimog EF Saragih IS Lin C-J. Adherence to Iron and folic acid supplementation (IFAS) intake among pregnant women: a systematic review meta-analysis. Midwifery. (2022) 104:103185. doi: 10.1016/j.midw.2021.103185, PMID: 34784576

[13] Nelson AJ Pagidipati NJ Bosworth HB. Improving medication adherence in cardiovascular disease. Nat Rev Cardiol. (2024) 21:417–29. doi: 10.1038/s41569-023-00972-1 38172243

[14] Cross AJ Elliott RA Petrie K Kuruvilla L George J. Interventions for improving medication-taking ability and adherence in older adults prescribed multiple medications. Cochrane Database Syst Rev. (2020) 2020:CD012419. doi: 10.1002/14651858.CD012419.pub2, PMID: 32383493 PMC7207012

[15] Chivu CM Tulchinsky TH Soares-Weiser K Braunstein R Brezis M. A systematic review of interventions to increase awareness, knowledge, and folic acid consumption before and during pregnancy. Am J Health Promot. (2008) 22:237–45. doi: 10.4278/06051566R2.1, PMID: 18421888

[16] Gomes F Bourassa MW Adu-Afarwuah S Ajello C Bhutta ZA Black R . Setting research priorities on multiple micronutrient supplementation in pregnancy. Ann N Y Acad Sci. (2020) 1465:76–88. doi: 10.1111/nyas.14267, PMID: 31696532 PMC7186835

[17] Esopo K Derby L Haushofer J. Interventions to improve adherence to antenatal and postnatal care regimens among pregnant women in sub-Saharan Africa: a systematic review. BMC Pregnancy Childbirth. (2020) 20:316. doi: 10.1186/s12884-020-02992-y, PMID: 32448165 PMC7245828

[18] Caut C Leach M Steel A. Dietary guideline adherence during preconception and pregnancy: a systematic review. Matern Child Nutr. (2020) 16:e12916. doi: 10.1111/mcn.12916, PMID: 31793249 PMC7083492

[19] Sendeku FW Azeze GG Fenta SL. Adherence to iron-folic acid supplementation among pregnant women in Ethiopia: a systematic review and meta-analysis. BMC Pregnancy Childbirth. (2020) 20:138. doi: 10.1186/s12884-020-2835-0, PMID: 32131751 PMC7057669

[20] Palmer MJ Machiyama K Woodd S Gubijev A Barnard S Russell S . Mobile phone-based interventions for improving adherence to medication prescribed for the primary prevention of cardiovascular disease in adults. Cochrane Database Syst Rev. (2021) 2021:CD012675. doi: 10.1002/14651858.CD012675.pub3, PMID: 33769555 PMC8094419

[21] Jansen MO Brown TR Xu KY Glowinski AL. Using digital technology to overcome racial disparities in child and adolescent psychiatry. J Am Acad Child Adolesc Psychiatry. (2022) 61:1211–7. doi: 10.1016/j.jaac.2022.03.013, PMID: 35358663 PMC9970009

[22] Lu HY Ding X Hirst JE Yang Y Yang J Mackillop L . Digital health and machine learning Technologies for Blood Glucose Monitoring and Management of gestational diabetes. IEEE Rev Biomed Eng. (2024) 17:98–117. doi: 10.1109/RBME.2023.3242261, PMID: 37022834 PMC7615520

[23] Lee VV Vijayakumar S Ng WY Lau NY Leong QY Ooi DSQ . Personalization and localization as key expectations of digital health intervention in women pre- to post-pregnancy. NPJ Digit Med. (2023) 6:183. doi: 10.1038/s41746-023-00924-6, PMID: 37775533 PMC10541409

[24] Godfrey A Powell D. UK funding agency launches digital health hubs: a new catalyst for change? NPJ Digit Med. (2024) 7:5. doi: 10.1038/s41746-023-00990-w, PMID: 38184701 PMC10771485

[25] Azizi Z Broadwin C Islam S Schenk J Din N Hernandez MF . Digital health interventions for heart failure Management in Underserved Rural Areas of the United States: a systematic review of randomized trials. J Am Heart Assoc. (2024) 13:e030956. doi: 10.1161/JAHA.123.030956, PMID: 38226517 PMC10926837

[26] Page MJ McKenzie JE Bossuyt PM Boutron I Hoffmann TC Mulrow CD . The PRISMA 2020 statement: an updated guideline for reporting systematic reviews. BMJ. (2021) 372:n71. doi: 10.1136/bmj.n71, PMID: 33782057 PMC8005924

[27] Higgins J Thomas J Chandler J Cumpston M Li T Page M . eds. Cochrane handbook for systematic reviews of interventions version 6.3 (updated February 2022). Cochrane (2022). Available at: www.training.cochrane.org/handbook.

[28] Palacio A Garay D Langer B Taylor J Wood BA Tamariz L. Motivational interviewing improves medication adherence: a systematic review and Meta-analysis. J Gen Intern Med. (2016) 31:929–40. doi: 10.1007/s11606-016-3685-3, PMID: 27160414 PMC4945560

[29] Wouters H Rhebergen D Vervloet M Egberts A Taxis K van Dijk L . Distinct profiles on subjective and objective adherence measures in patients prescribed antidepressants. Drugs. (2019) 79:647–54. doi: 10.1007/s40265-019-01107-y, PMID: 30941607 PMC6483946

[30] Wan X Wang W Liu J Tong T. Estimating the sample mean and standard deviation from the sample size, median, range and/or interquartile range. BMC Med Res Methodol. (2014) 14:135. doi: 10.1186/1471-2288-14-135 25524443 PMC4383202

[31] Sterne JAC Egger M. Funnel plots for detecting bias in meta-analysis: guidelines on choice of axis. J Clin Epidemiol. (2001) 54:1046–55. doi: 10.1016/S0895-4356(01)00377-8 11576817

[32] Sterne JAC Savović J Page MJ Elbers RG Blencowe NS Boutron I . RoB 2: a revised tool for assessing risk of bias in randomised trials. BMJ. (2019) 366:l4898. doi: 10.1136/bmj.l4898 31462531

[33] Balshem H Helfand M Schünemann HJ Oxman AD Kunz R Brozek J . GRADE guidelines: 3. Rating the quality of evidence. J Clin Epidemiol. (2011) 64:401–6. doi: 10.1016/j.jclinepi.2010.07.015 21208779

[34] Sontakke P Dwidmuthe KS Kawathalkar A Bhalerao A. Effect of Mobile phone call reminders with standard therapy versus standard therapy alone on compliance with Iron supplementation in antenatal women with Iron deficiency Anemia: a randomized controlled trial. Cureus. (2022) 14:e29501. doi: 10.7759/cureus.29501, PMID: 36299926 PMC9588298

[35] Ramachandran R Dash M Adaikaladorai FC Aridass J Zachariah B Manoharan B. Effect of individual nutrition education on perceptions of nutritional iron supplementation, adherence to iron-folic acid intake and Hb levels among a cohort of anemic south Indian pregnant women. J Matern Fetal Neonatal Med. (2023) 36:2183749. doi: 10.1080/14767058.2023.2183749, PMID: 36852425

[36] Sharma S Smitha MV Balakrishnan D. Telephonic intervention to combat non-adherence to oral iron-folic acid supplementation in pregnancy: a randomized controlled trial. Eur J Obstet Gynecol Reprod Biol X. (2023) 20:100235. doi: 10.1016/j.eurox.2023.100235, PMID: 37736306 PMC10509657

[37] Arifah I Pambarep TSA Khoiriyah L Kusumaningrum TAI Werdani KE Ngadiyono NP. Effectiveness of daily educational message on pregnancy anemia prevention behavior and knowledge: a pilot randomized controlled trial. J Educ Health Promot. (2023) 12:296. doi: 10.4103/jehp.jehp_108_23, PMID: 37849860 PMC10578550

[38] Elsharkawy NB Abdelaziz EM Ouda MM Oraby FA. Effectiveness of health information package program on knowledge and compliance among pregnant women with Anemia: a randomized controlled trial. Int J Environ Res Public Health. (2022) 19:2724. doi: 10.3390/ijerph19052724, PMID: 35270420 PMC8910269

[39] Berhane A Belachew T. Effect of picture-based health education and counselling on knowledge and adherence to preconception Iron-folic acid supplementation among women planning to be pregnant in eastern Ethiopia: a randomized controlled trial. J Nutr Sci. (2022) 11:e58. doi: 10.1017/jns.2022.51, PMID: 35912303 PMC9305079

[40] Ariyani NW Wirawan IMA Pinatih GNI Kusuma AANJ. The effect of an application-based educational intervention with a social cognitive theory model on pregnant women in Denpasar, Bali, Indonesia: a randomized controlled trial. Osong Public Health Res Perspect. (2022) 13:153–61. doi: 10.24171/j.phrp.2021.0209, PMID: 35538687 PMC9091633

[41] Abd Rahman R Idris IB Md Isa Z Abd RR. The effectiveness of a theory-based intervention program for pregnant women with anemia: a randomized control trial. PLoS One. (2022) 17:e0278192. doi: 10.1371/journal.pone.0278192, PMID: 36473006 PMC9725169

[42] Abujilban S Hatamleh R Al-Shuqerat S. The impact of a planned health educational program on the compliance and knowledge of Jordanian pregnant women with anemia. Women Health. (2019) 59:748–59. doi: 10.1080/03630242.2018.1549644, PMID: 30596538

[43] Khorshid MR Afshari P Abedi P. The effect of SMS messaging on the compliance with iron supplementation among pregnant women in Iran: a randomized controlled trial. J Telemed Telecare. (2014) 20:201–6. doi: 10.1177/1357633X14533895, PMID: 24803276

[44] Karwa S Jahnke H Brinson A Shah N Guille C Henrich N. Association between doula use on a digital health platform and birth outcomes. Obstet Gynecol. (2024) 143:175–83. doi: 10.1097/AOG.0000000000005465, PMID: 38052036 PMC10789380

[45] Brammall BR Garad RM Teede HJ Harrison CL. Evaluating preconception health and behaviour change in Australian women planning a pregnancy: the OptimalMe program, a digital healthy lifestyle intervention with remotely delivered coaching. Nutrients. (2024) 16:155. doi: 10.3390/nu16010155, PMID: 38201984 PMC10780803

[46] Lazarevic N Pizzuti C Rosic G Bœhm C Williams K Caillaud C. A mixed-methods study exploring women’s perceptions and recommendations for a pregnancy app with monitoring tools. NPJ Digit Med. (2023) 6:50. doi: 10.1038/s41746-023-00792-0, PMID: 36964179 PMC10036977

[47] Goodday S Karlin D Suver C Friend S. The post-roe political landscape demands a morality of caution for Women’s health. J Med Internet Res. (2022) 24:e41417. doi: 10.2196/41417, PMID: 36264611 PMC9634512

[48] Moise IK Ivanova N Wilson C Wilson S Halwindi H Spika VM. Lessons from digital technology-enabled health interventions implemented during the coronavirus pandemic to improve maternal and birth outcomes: a global scoping review. BMC Pregnancy Childbirth. (2023) 23:195. doi: 10.1186/s12884-023-05454-3, PMID: 36941565 PMC10026210

[49] Zingg A Singh T Franklin A Ross A Selvaraj S Refuerzo J . Digital health technologies for peripartum depression management among low-socioeconomic populations: perspectives from patients, providers, and social media channels. BMC Pregnancy Childbirth. (2023) 23:411. doi: 10.1186/s12884-023-05729-9, PMID: 37270494 PMC10239590

