# Supplementary material for: Digital versus non-digital health interventions to improve iron supplementation in pregnant women: a systematic review and meta-analysis
Source: Front Med (Lausanne). 2024 May 30;11:1375622. doi: 10.3389/fmed.2024.1375622 (PMC11173591; doi:10.3389/fmed.2024.1375622)
Supplement: SUPPLEMENTARY FIGURE S1 — Funnel plot of publication bias for objective adherence rate. [file Data_Sheet_1.docx]

**Supplementary Table S1. Example search strategy.**

The search strategy for PubMed was shown below. It was adapted for the other databases.

| **Pubmed**  (pregnan*[Title/Abstract] OR gravid[Title/Abstract] OR gestation*[Title/Abstract] OR maternal[Title/Abstract] OR "Pregnancy"[Mesh]) AND (digital[Title/Abstract] OR "electronic intervention*"[Title/Abstract] OR "internet"[Title/Abstract] OR "mobile phone"[Title/Abstract] OR "smartphone"[Title/Abstract] OR "app"[Title/Abstract] OR "E-health"[Title/Abstract] OR "online"[Title/Abstract] OR "text message"[Title/Abstract] OR "multimedia message"[Title/Abstract] OR "mobile application"[Title/Abstract] OR "social media"[Title/Abstract] OR "email"[Title/Abstract] OR "cell phone"[Title/Abstract] OR "mhealth"[Title/Abstract] OR "m-health"[Title/Abstract] OR "telehealth"[Title/Abstract]  OR "twitter"[Title/Abstract] OR "facebook"[Title/Abstract] OR "iphone"[Title/Abstract] OR "ipad"[Title/Abstract] OR "webinar"[Title/Abstract] OR "electronic aids"[Title/Abstract] OR "ehealth"[Title/Abstract] OR "web"[Title/Abstract] OR "website"[Title/Abstract] OR "computer"[Title/Abstract] OR "sms"[Title/Abstract] OR "msm"[Title/Abstract] OR "android"[Title/Abstract]) AND ("iron"[MeSH Terms] OR "iron"[All Fields]) AND ("patient compliance"[MeSH Terms] OR "patient participation"[MeSH Terms] OR "complian*"[MeSH Terms] OR "motivation"[MeSH Terms] OR "cooperative behavior"[MeSH Terms] OR "adhere*"[Title/Abstract] OR "complian*"[Title/Abstract] OR "noncomplian*"[Title/Abstract] OR "nonattend*"[Title/Abstract] OR "attendance"[Title/Abstract] OR "motivat*"[Title/Abstract] OR "complying"[Title/Abstract]) | **Adapted for:**  Cochrane, Embase,  Web of Science, and Scopus. |
| --- | --- |


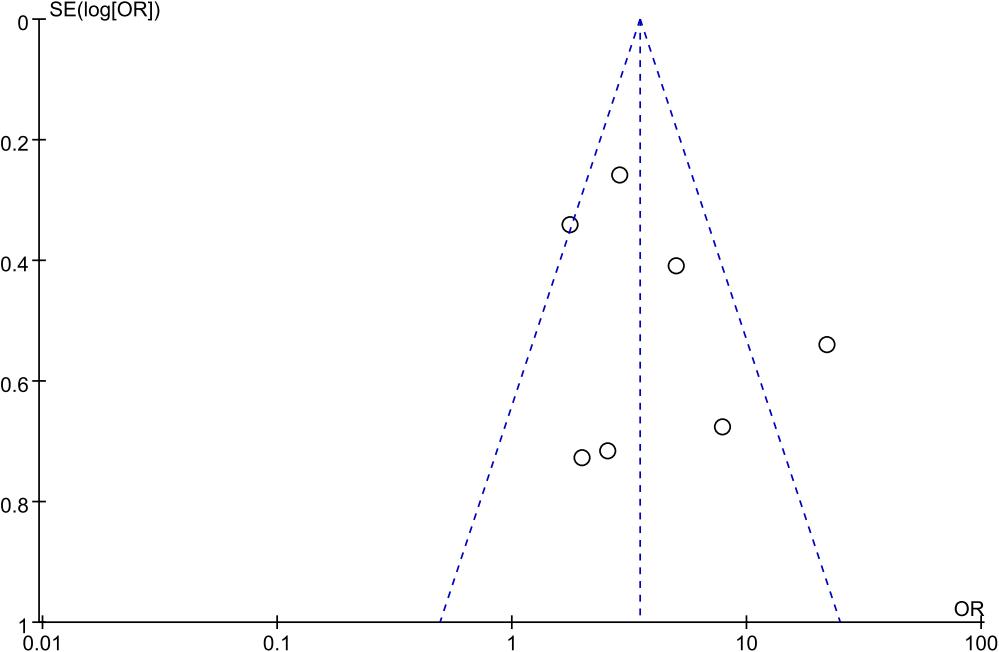


**Supplementary Figure S1. The funnel plots of objective adherence rate.**


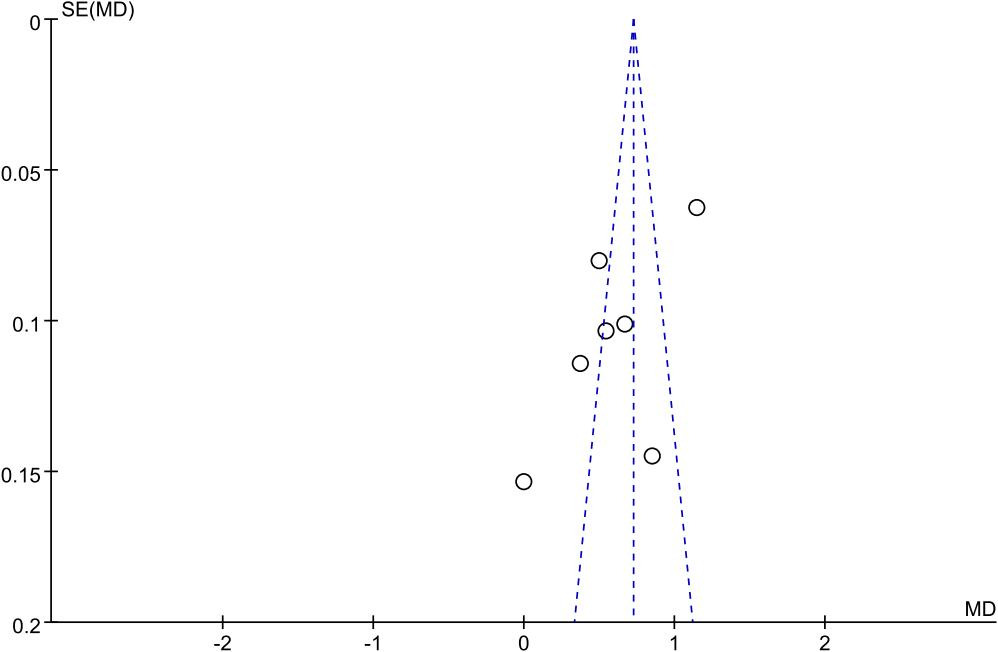


**Supplementary Figure S1. The funnel plots of hemoglobin level.**
